# Supplementary material for: A Pilot Study to Examine Exposure to Residential Radon in Under-Sampled Census Tracts of DeKalb County, Georgia, in 2015
Source: Int J Environ Res Public Health. 2017 Mar 22;14(3):332. doi: 10.3390/ijerph14030332 (PMC5369167; doi:10.3390/ijerph14030332)
Supplement: Supplementary file 1 [file ijerph-14-00332-s001.pdf]

Supplementary Materials: A Pilot Study to Examine Exposure to Residential Radon in Under-Sampled Census Tracts of DeKalb County, Georgia, in 2015

Christine Stauber *, Dajun Dai, Sydney Chan, Jeremy Diem, Scott Weaver and Richard Rothenberg

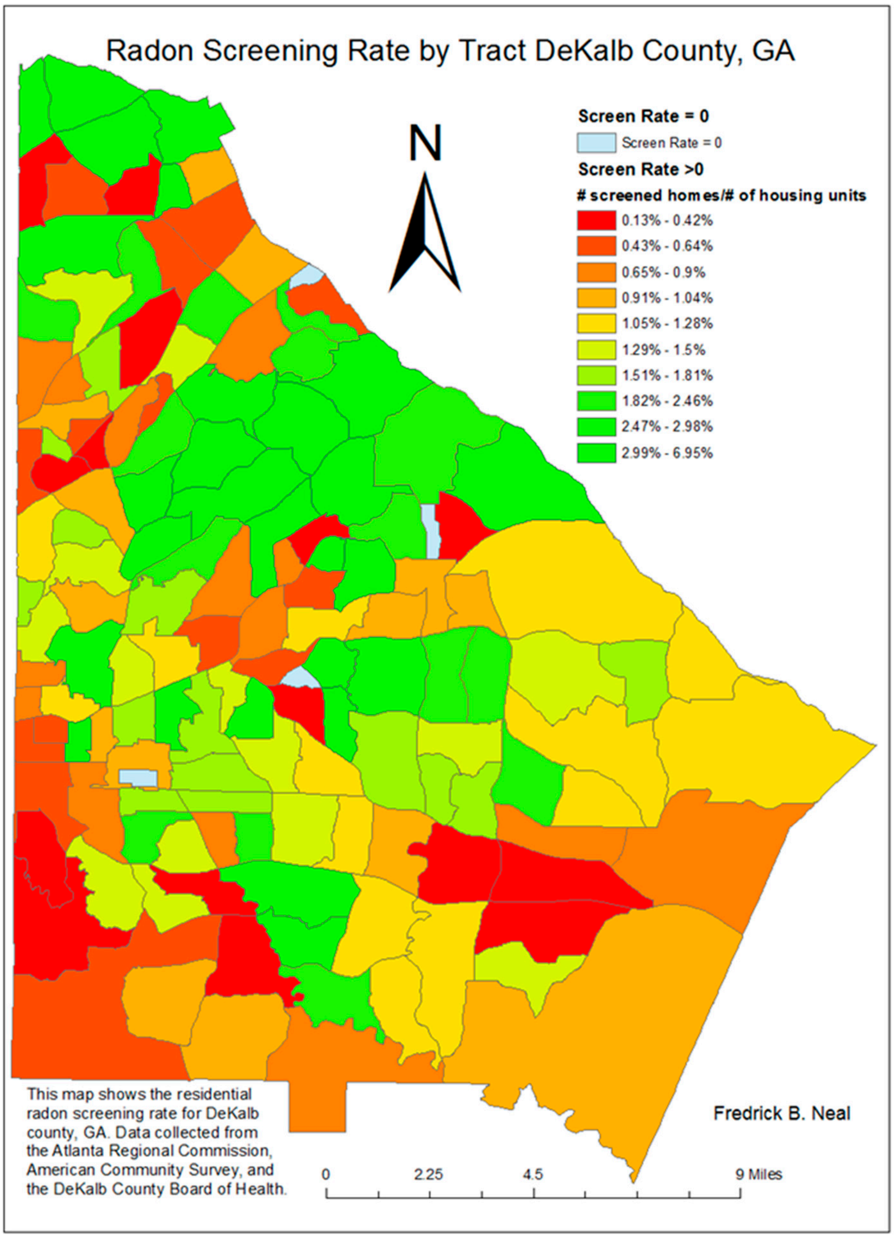

Figure S1. Radon screening prevalence in DeKalb County, GA.

© 2017 by the authors; licensee MDPI, Basel, Switzerland. This article is an open access article distributed under the terms and conditions of the Creative Commons by Attribution (CC-BY) license (http://creativecommons.org/licenses/by/4.0/).
